# Chemical Modification of Butyl Rubber with Maleic Anhydride via Nitroxide Chemistry and Its Application in Polymer Blends

**DOI:** 10.3390/polym9020063

**Published:** 2017-02-20

**Authors:** José Bonilla-Cruz, Brenda Hernández-Mireles, Ricardo Mendoza-Carrizales, Luis A. Ramírez-Leal, Román Torres-Lubián, Luis F. RamosdeValle, Donald R. Paul, Enrique Saldívar-Guerra

**Affiliations:** 1 Centro de Investigación en Química Aplicada (CIQA), Blvd. Enrique Reyna 140, Saltillo 25294, Mexico; jose.bonilla@cimav.edu.mx (J.B.-C.); Brenda_caesio@hotmail.com (B.H.-M.); ricardo.mendoza@ciqa.edu.mx (R.M.-C.); roman.torres@ciqa.edu.mx (R.T.-L.); luis.ramos@ciqa.edu.mx (L.F.R.V.); 2 Advanced Functional Materials & Nanotechnology Group, Centro de Investigación en Materiales Avanzados (CIMAV-Unidad Monterrey), Av. Alianza Norte 202, Autopista Monterrey-Aeropuerto Km 10, PIIT, Apodaca 66628, Mexico; luis.ramirez.leal@gmail.com; 3 Department of Chemical Engineering, University of Texas at Austin, 1 University Station C0400 Austin, Austin, TX 78712-0231, USA; drp@che.utexas.edu

**Keywords:** polymer grafting, butyl rubber, nitroxide-mediated polymerization, polymer nanocomposite, polymer blend

## Abstract

Butyl rubber (isobutylene–isoprene–rubber, IIR) was functionalized in solution with a nitroxide moiety taking advantage of the unsaturations present in the isoprene units of IIR, and was further grafted with maleic anhydride (MA) or styrene–MA (SMA) to produce IIR-*g*-MA and IIR-*g*-SMA. In one of the functionalization techniques used, the molecular structure of the IIR was preserved as the chain-breaking reactions are prevented from occurring. The resulting graft copolymers were tested as compatiblizers/impact modifiers blended with Nylon-6, and one of them was preliminarily tested as a coupling agent in the preparation of nanocomposites of IIR and an organo-clay. Blends of PA-6/IIR-*g*-MA exhibited a significant increase in impact resistance at increasing loads of the modified IIR, as well as a good rubber particle dispersion in the polyamide matrix. On the other hand, the performance of IIR-*g*-SMA as an impact modifier of PA, or as a coupling agent in the preparation of rubber-organoclay nanocomposites, is marginal.

## 1. Introduction

Grafted and functionalized polymers are very attractive materials from both the scientific and the industrial points of view due to their potential applications as compatibilizers [1], adhesives [2], surfactants, etc. Grafting side chains or single entities containing polar groups can impart polarity to polymers that are otherwise nonpolar. For example, polyethylene and polypropylene are modified with maleic anhydride (MA) via reactive processing [3,4,5], although little control of the level and the homogeneity of the functionalization can be achieved by this process.

In this work a procedure that we have developed for controlled functionalization and grafting with MA and styrene–MA using nitroxide chemistry (more details below) is applied to butyl rubber, or IIR. This is a more challenging system than others previously studied by us due to the fact that the unsaturations present in IIR are only a small percentage (less than 2% molar) of the rubber chain and these unsaturations represent the labile sites of the molecules at which nitroxide moieties are attached. The resulting modified IIR is preliminarily tested as a compatibilizer in polymer blends of IIR with nylon 6 (labeled here as PA-6) in order to study their mechanical behavior and as a coupling agent in nanoclay dispersion in a butyl rubber matrix.

### Background

Butyl rubber (IIR) is the copolymer of isobutylene and a small amount of isoprene, which is commercially produced by cationic polymerization. It was first commercialized in the early 1940s; its main attributes are excellent gas barrier and good flex properties, resulting from low levels of unsaturated units between long polyisobutylene segments [6]. On the other hand, IIR grafted with reactive groups (e.g., MA) has only been studied to a limited extent [7,8,9,10,11,12]; also, very little is known about compatibilization studies for blends of butyl rubber with other polymers that can result in materials with better overall properties.

Butyl rubber has been modified by several research groups. In a 1972 patent [8] from Esso Research Engineering Company, a method for inserting ionic species into the butyl rubber structure was disclosed: First, a halogenated butyl rubber is dissolved in a suitable organic solvent in the presence of a base or metal hydroxide to produce a dehydrohalogenated butyl rubber (dehydrohalogenation results in a butyl rubber having conjugated double bonds in the linear backbone). Then, an ethylenically unsaturated compound (maleic anhydride, among others) is added. The reaction between maleic anhydride and the conjugated double bonds is carried out spontaneously by a Diels–Alder cycloaddition, producing a bicyclic anhydride derivative with the same molecular weight as the starting polymer. In a related more recent and promising technique [9], epoxidized IIR is first subjected to acid catalyzed ring-opening followed by elimination to form different dienes depending on the reaction conditions used; then, Diels–Alder cyclo-addition on the exo-diene is used to prepare a variety of multifunctional graft copolymers of IIR and even a heat-reversible cross-linked rubber.

Also, maleated butyl rubber has been produced using halogenated butyl rubber and maleic anhydride in the presence of: (i) an acid acceptor and (ii) a monofunctional hindered monoamine and/or monofunctional hindered phenol [10]. Another route to produce maleated IIR starting from its brominated derivative was proposed in a 2007 paper as a route for the graft modification of IIR. The authors carried out selective dehydrohalogenation to produce an exoconjugated diene, which was further reacted with MA via a Diels–Alder cycloaddition; this product was then used as an intermediate for further alcoholysis with alcohol-terminated polyethylene (PE), which leads to IIR-*g*-PE [11]. In a 2009 patent [12], IIR was modified in the presence of a free radical initiator and maleic anhydride (in one of the claims) to obtain a maleated liquid IIR with reduced molecular weight with respect to the source IIR material. The treatment of the IIR with a free radical initiator results in a loss of molecular weight by chain degradation. In another work [13], butyl rubber was treated with electron beam radiation in the presence of butyl acrylate or methyl methacrylate in order to graft acrylic homopolymers to the rubber backbone; however, it is difficult to control the level of grafting by this technique and chain degradation by scission is also reported by the authors.

Perova et al. [14] reported the maleation of butyl rubber in a mixing chamber in the presence of a peroxide radical initiator and maleic anhydride above 150 °C. Grafting (maleation) of the rubber occurs simultaneously with chain degradation. The resulting material is studied in applications as a nonfreezing sealant.

Butyl rubber has been also grafted with polyethylene oxide (PEO) [15]. First, the double bonds of the isoprene units were converted to epoxides using m-chloroperoxy-benzoic acid. Next, the epoxidized IIR was reacted with HCl in toluene to promote the ring-opening of the epoxide, resulting in the formation of alkene and hydroxyl groups. Later, this product was reacted with 4-nitrophenyl chloroformate in the presence of triethylamine and toluene to produce activated carbonates. Finally, this polymer was reacted with poly(ethylene glycol) monomethyl ether (m-PEG) with a molecular weight of 2000 g/mol by substitution of the 4-nitrophenol leaving groups, leading to a graft copolymer.

Finally, bromination is a standard chemical procedure used to modify butyl rubber at the industrial scale. It involves a substitution reaction of molecular bromine with the isoprene units of butyl rubber in solution, with evolution of hydrogen bromide as a side product [16]. The resulting brominated IIR is a more polar material exhibiting faster cure rates than the non-brominated IIR.

From the brief review of IIR modification discussed above, it is clear that, for the case of maleation, most of the known procedures end up with the breakage of polymer chains and reduction of molecular weight and material strength, which results in, for example, reduced adhesive and cohesive strength when the modified IIR is used in an adhesive formulation [15].

In a 2003 paper, Parent and collaborators [17] used several non-polymeric model compounds resembling the structures of polyethylene and polybutadiene to test the viability of reduced-crosslinking maleation of these substrates, mediated by the use of different nitroxyl radicals. They concluded that the grafting of a single unit of maleic anhydride should be viable in polybutadiene, and additional mechanistic and kinetic insight, which is discussed afterwards, was also provided.

In a previous work of our group we successfully grafted unsaturation-containing rubbers such as polybutadiene, polyisoprene, and EPDM by a two-step procedure. In a first step a solution of the rubber is heated in the presence of a radical initiator and a nitroxide stable free radical such as 2,2,6,6 tetramethyl piperidine 1-oxyl (TEMPO) or 4-hydroxy TEMPO (OH-TEMPO) in order to produce a nitroxide-functionalized rubber; in a second step, this material is further heated in solution in the presence of one or two vinyl monomers (e.g., styrene and or MA) in order to create polymeric grafts or functional groups containing the monomer units covalently bonded to the rubber backbone [18,19,20]. This procedure has the advantage of providing better control of the number of grafts or functional groups as well as the length of the grafts by optimizing the ratio of nitroxide to number of unsaturations in the rubber substrate. Improved control of these structural features in the modified rubber can be advantageous for the development of applications in which some form of modified IIR is used as an adhesive [15], a compatibilizer in blends with polar polymers (e.g., polyamide) for improved impact resistance [21] or a coupling agent for composite formulations with nanoclays [22], among others.

## 2. Materials and Methods

### 2.1. Materials

Butyl rubber (IIR: Exxon Butyl 286, *M*_n_ = 192,095 Da., PD = 2.4, unsaturations = 1.8 wt % (1.5 mol %), kindly provided by Exxon Mobil Chemical Company, Spring, TX, USA), Nylon 6 (PA-6: Capron B135WP, *M*_n_ = 29,300 Da., MFI = 1.2 g/10 min provided by Honeywell International Inc., Morris Plains, NJ, USA), 4-hydroxy-2,2,6,6-tetramethylpiperidine-1-oxyl free radical (4-hydroxy-TEMPO), 2,5-*bis*(*tert*-butylperoxy)-2,5-dimethylhexane (DTBH), styrene (St, +99.9%), maleic anhydride (MA, +99.9%), toluene, methanol, chloroform, phosphotungstic acid (PTA), benzyl alcohol (BA), *N*,*N*-dimethylformamide (DMF), and deionized water (H_2_O DI) were purchased from Sigma-Aldrich Química, Toluca, México, and were used as received. Styrene monomer was distilled twice under a vacuum in the presence of calcium hydride.

### 2.2. Instrumentation

^1^H nuclear magnetic resonance (NMR) spectra were recorded using a Fourier Transform NMR (FT-NMR) Jeol Eclipse 300 instrument (Jeol Company Inc., Peabody, MA, USA) at 300 MHz in CDCl_3_ at room temperature. For the analysis of nitroxide holding groups by ^1^H, 20 mg of sample were used to collect the spectrum with 100 scans. Fourier Transform Infrared (FTIR) spectra were recorded with a Nexus 470 spectrometer (Thermo Fisher Scientific, Waltham, MA, USA) of 4000–400 cm^−1^ using 32 scans and 4 cm^−1^ of resolution. Molecular weights, relative to polystyrene standards, were determined by gel permeation chromatography (GPC) using ultrastyragel columns ((1 K–40 K), 10^3^ Å, (40 K–4 M), 10^5^ Å, and (400 K–40 M), 10^6^ Å) with tetrahydrofuran (THF) at 40 °C as solvent and a flow rate of 1 mL/min, coupled to a Waters 410 differential refraction index refractometer and ultraviolet-visible detector (Waters Corporation, Milford, MA, USA). Scanning–transmission electron microscopy (STEM) micrographs were obtained by Scanning Electron Microscopy (SEM) in transmission mode and were performed using a FEI Nano SEM 200 (Fei Company, Hillsboro, OR, USA) at an accelerating voltage of 15 kV. Also, some samples were observed using a transmission electron microscopy (TEM) using a JEOL 2010F (Jeol Company Inc., Peabody, MA, USA) at an accelerating voltage of 120 kV. STEM & TEM samples for morphology characterization were cryogenically cut into ultrathin sections (30 to 50 nm thick) with a diamond knife at a temperature of −40 °C with a RMC PowerTome Ultramicrotome XL (Boeckeler Instruments, Tucson, AZ, USA). These sections were taken from the central part of a tensile bar normal to the direction of flow.

### 2.3. Functionalization of IIR with Nitroxide Moieties at Lab Scale: IIRF1

Twenty grams of IIR (1.8 wt % of isoprenic portion = 0.36 g = 0.0053 mol) and 0.901 g of 4-hydroxy-TEMPO (0.0052 mol, 4.5 wt % with respect to the rubber) were dissolved in 60 mL of toluene in a 100-mL glass reactor heated by a jacket and equipped with a magnetic stirrer and condenser. The reaction system was purged using high-purity Argon gas for 10 min. After that, the solution was heated to 135 °C. When the reaction mixture reached 135 °C, a solution of initiator DTBH (0.55 g = 0.0019 mol in 20 mL of toluene) was added dropwise in order to prevent the cross-linking reaction of the rubber. A molar relationship nitroxide:initiator 2.7:1 was used. The functionalization reaction proceeded for 7 h under inert atmosphere. The functionalized polymer with nitroxide moieties (IIRF1) was purified three times by precipitation in methanol and dissolution in chloroform in order to remove unreacted initiator, free nitroxide, and toluene remains. A light brown product was recovered and dried to remove the residual methanol using a vacuum at room temperature for 48 h and was characterized by ^1^H NMR and GPC.

### 2.4. Synthesis of Model Compatibilizer IIR-g-MA at Lab Scale

Six grams of IIR-*g*-Nitroxide (IIRF1) were dissolved in 80 mL of toluene in a 100-mL glass reactor having a jacket and equipped with magnetic stirrer and condenser, in the presence of 0.5 g (0.005 mol, 3 wt % with respect to the rubber) of MA. The reaction system was purged using high-purity argon gas for 10 min and heated at 125 °C. The functionalization reaction (substitution of nitroxide moieties by MA groups) was achieved in 2 h. The functionalized polymer with maleic anhydride moieties (IIR-*g*-MA) was purified three times by precipitation in methanol and dissolution in chloroform. The IIR-*g*-MA model compatibilizer was recovered and was dried to remove residual methanol using vacuum at room temperature for 48 h and was then characterized by ^1^H NMR and GPC.

### 2.5. Functionalization of IIR with Nitroxide Moieties at a Large Scale: IIRF2

Two hundred eighty grams of IIR (1.8 wt % of isoprenic portion = 5.04 g = 0.074 mol) and 38.23 g of 4-hydroxy-TEMPO (0.22 mol, 13.6 wt % with respect to the rubber) were dissolved in 6000 mL of toluene, in the presence of 23.66 g (0.08 mol) of initiator DTBH in a five-gallon stainless steel Parr (Parr Instrument Company, Moline, IL, USA) pressure reactor, heated by a jacket and equipped with a mechanical stirrer and serpentine cooler. The reactor was closed and purged using high-purity argon gas for 10 min. After that, the reaction system was heated to 135 °C. The molar ratio of nitroxide to initiator and the reaction time, as well as the purification procedure, were the same as those used in the lab scale. A light brown product was recovered, dried to remove the residual methanol using a vacuum at room temperature for 48 h, and characterized by ^1^H NMR, FTIR, and GPC. This procedure was also used at the lab scale to produce smaller batches of the functionalized rubber.

### 2.6. Synthesis of Compatibilizer IIR-g-SMA at a Large Scale

Next, 122.4 g of IIR-*g*-Nitroxide (IIRF2) were dissolved in 6000 mL of toluene in a five-gallon stainless steel Parr pressure reactor, heated by a jacket and equipped with mechanical stirrer and serpentine cooler, in the presence of 865.8 g (8.37 mol) of St monomer, 43 g of MA (0.43 mol, 35.1 wt % with respect to the rubber), and 4 mL of DMF to improve the MA dilution. The reaction system was purged using high-purity argon gas during 10 min and heated at 125 °C for 4 h for the production of short grafts (SG IIR-*g*-SMA) or for 6 h for the production of long grafts (LG IIR-*g*-SMA). The functionalized polymer with controlled grafts (IIR-*g*-SMA) was purified three times by precipitation in methanol and dissolution in chloroform. IIR-*g*-SMA compatibilizer was recovered, dried to remove the residual methanol using a vacuum at room temperature for 48 h, and characterized by ^1^H NMR and GPC.

### 2.7. Melt Processing

Several batches of 3.6 g of PA-6/IIR, PA-6/IIR-*g*-MA, and PA-6/IIR-*g*-SMA (LG) were prepared in a 5-mL DSM (Xplore Instruments BV, Sittard, The Netherlands) microcompounder (Table 1 summarizes the experimental design), which consists of a vertical barrel with two conical screws (screw diameter range: 0.43 cm to 1 cm, screw length: 10.75 cm). The residence time used was 15 min. The screw speed and the temperature in the microcompounder were fixed at 80 rpm and 290 °C, respectively. The same procedure was used to obtain a “control sample” using PA-6/IIR (80/20 wt %). However, in this case the conditions used in the microcompounder were 240 °C at 80 rpm for 2 min. The extruder blends were transferred to a DSM bench-top microinjection molder in order to obtain specimens (bars) of 7.3 cm × 1 cm × 0.32 cm. The molding and cylinder barrel temperature used were 130 and 292 °C, respectively. The injection pressure used was 40 psi for 1 min.

### 2.8. Preparation of Clay Nanocomposites

Preliminary testing of IIR-*g*-SMA (SG and LG) as coupling agent for the preparation of nanocomposites was made. To this end, butyl rubber with and without compatibilizer (1–8 parts phr) and an organo-clay (Cloisite 20A, Southern Clay Products Inc., Gonzales, TX, USA), 4–15 parts phr) were mixed in a 75 cc Brabender (Brabender Technologie GMBH & CO, Duisburg, Germany) type mixer at 60 rpm and 110 °C. The resulting materials were then analyzed by STEM to examine the level of dispersion. Samples for tensile testing were prepared in an 85-cc Brabender mixer using a standard vulcanization procedure at 160 °C for 22 min. Mechanical testing was run in a Universal Instron (Instron, Norwood, MA, USA) machine at a speed of 6 in/min at 23 °C and 50% relative humidity.

### 2.9. Microtoming Procedure

Ultra-thin sections (30–50 nm) of the material were cryogenically microtomed using a RMC PowerTome Ultramicrotome XL equipped with a MicroStar (Microstar Technologies, Huntsville, TX, USA) diamond knife (length 2.5 mm) at −60 °C. In a typical procedure, the specimens (bars) were cut from the plane perpendicular to the flow direction to obtain small rectangular pieces which were glued onto mounting cylinders of epoxy resin. A microtome Reichert-Jung (Reichert Technologies, Depew, NY, USA) Ultracut E was used in order to make rectangular pieces which were cut with stainless steel knives in pyramidal form preserving a surface at the tip to the pyramid of 0.2 × 0.2 mm^2^. Finally, the tip of the pyramid was progressively trimmed to obtain ultra-thin layers of about 30–50 nm in thickness.

### 2.10. Staining Procedure

The polyamide phase present in the samples was stained selectively using an aqueous solution of phosphotungstic acid (PTA, 2 wt %), benzyl alcohol (BA, 2 wt %), and deionized water (H_2_O DI, 96 wt %). PTA stains functional groups (amide present in the nylon) whereas BA improves the PTA diffusion into the sample [23]. In a typical procedure, 0.15 mL of PTA/BA/H_2_O and 0.15 mL of deionized water (DI) were placed in a staining dish in individual holes. The cupper grid containing the microtomed slices (dark face) was placed onto the solution of PTA/BA/H_2_O with the dark face down for 1 h. After that, the floating grid was picked up and placed onto DI water for 30 min (dark face down). Finally, the grid was removed and dried with filter paper with the dark face up.

## 3. Results and Discussions

### 3.1. Synthesis Procedures and Possible Mechanisms

**Scheme 1:**
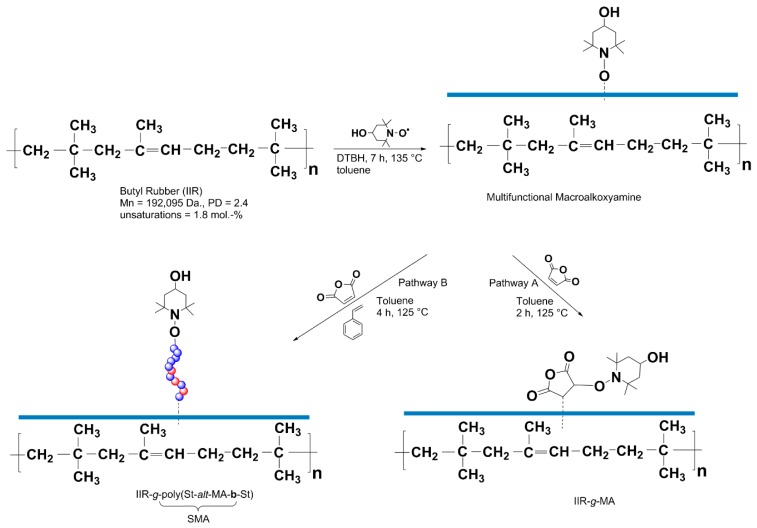
Functionalization of butyl rubber with nitroxide groups and its use in the synthesis of model compatibilizers: IIR-*g*-MA and IIR-*g*-SMA. The functionalization step involves a hydrogen abstraction, which is denoted with the symbol “–H” at the right of the multifunctional macroalkoxyamine structure (see explanation in the text). The two colors in the grafted chain represent the two types of monomeric units (MA, red, and styrene, blue).

Two synthetic pathways were used in this work in order to obtain model compatibilizers based on butyl rubber (IIR), as displayed in Scheme 1. The upper part of Scheme 1 schematically shows the single procedure by which the polymeric multifunctional macroalkoxyamine is synthesized by heating IIR at 135 °C in solution, in the presence of a combination of nitroxide and a *tert*-butoxy radical source (bimolecular process). IIR functionalized with 4-hydroxy-TEMPO moieties along its backbone was produced using two initiator addition procedures and scales. The multifunctional macroalkoxyamines obtained were identified as IIRF1 and IIRF2. In the first case, in order to obtain IIRF1 at the lab scale, the initiator was added dropwise to a solution of IIR/nitroxide once the reaction temperature (135 °C) was reached; on the other hand, in order to functionalize IIRF2 at a larger scale, the initiator and nitroxide were dissolved into the IIR solution, and the reaction system was heated progressively up to 135 °C; it was considered that this second procedure would be closer to standard industrial practice.

The peroxidic initiator DTBH was used as a *tert*-butoxy radical source, because this kind of oxygen-centered radical shows relatively high regio-specificity in reactions with carbon–carbon double bonds; they exhibit a propensity for hydrogen atom abstraction (as opposed to addition), which is enhanced at higher temperatures [4] or in the presence of aromatic solvents [24]. Thus, the *tert*-butoxy radicals can abstract an allylic hydrogen from the isoprenic portion in the IIR, as discussed before by our group regarding the functionalization of polyisoprene with nitroxide groups [20]. It has been reported that hydrogen abstraction by *tert*-butoxy radicals, which are very reactive, is controlled by entropic rather than enthalpic factors [25]; therefore, in this case, the abstraction is more likely to occur at the primary hydrogens instead of the secondary ones, although both instances are possible. It is postulated that hydrogen abstraction produces a carbon-centered radical (in the isoprenic portion) that can be quickly capped by the stable free radical 4-hydroxy-TEMPO, thus producing a multifunctional macroalkoxyamine. Using NMR, we attempted to define the preferred site of hydrogen abstraction and the exact mechanism of attachment of the nitroxide moiety to the IIR backbone but, due to the low proportion of isoprenic units in IIR, it was not possible to obtain conclusive evidence of these mechanistic aspects by this means or by the functionalization reaction efficiency. Therefore, only a model is shown for this reaction step in Scheme 1. Additional work is ongoing in our group to further elucidate this issue.

The addition procedures of the initiator in the functionalization process have a strong influence on the molecular weights and polydispersities of the resulting functionalized butyl rubber, as will be demonstrated below using gel permeation chromatography.

A model compatibilizer named for simplicity IIR-*g*-MA (although it is likely an IIR-*g*-succinic anhydride due to the loss of the double bond in the MA moiety) can be obtained by following Pathway A. Under this route, the macroalkoxyamine IIRF1 in the presence of maleic anhydride (MA, 3 wt % with respect to IIRF1) and toluene as solvent, is heated up to 125 °C to promote its homolytic cleavage, obtaining thus a stable free radical 4-hydroxy-TEMPO and a carbon-centered radical (in the isoprenic portion). Latter, this radical reacts with the double bond of MA, producing a covalent bond between MA and IIR, and a second carbon-centered radical that is capped by 4-hydroxy-TEMPO. This assumption is based on the fact that the homopropagation of MA monomer is extremely difficult, at least under relatively mild reaction conditions [26].

**Scheme 2:**
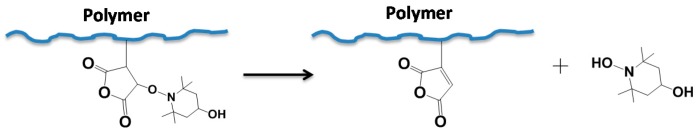
Disproportionation reaction of the polymer-*g*-succinic anhydride to yield the polymer-*g*-maleic anhydride and hydroxylamine.

Additionally, it has been reported that, depending on the reaction conditions [17,27], succinic alkoxyamines similar to that formed by the grafting reaction of the functionalized polymer with MA in Scheme 1 tend to undergo a disproportionation reaction, leading to a true maleic anhydride graft and an hydroxylamine (Scheme 2). In our case both graft structures can be obtained, one with the succinic alkoxyamine and the other with the maleic anhydride. However, as discussed below, it was not possible to discern the extent of the disproportionation reaction and therefore both structures are possible (for simplicity, they will be referred to generically as IIR-*g*-MA).

Also, a second model compatibilizer based on IIR possessing controlled grafts of poly[(styrene-alt-maleic anhydride)-*block*-styrene] (labeled IIR-*g*-SMA) was produced following Pathway B. In this case, the macroalkoxyamine IIRF2 was dissolved in toluene in the presence of St monomer (in a 7:1 St:IIRF2 wt ratio), MA (~35 wt % with respect to IIRF2) and a small amount of DMF (4 mL) to improve the MA dissolution. At 135 °C the homolytic fission of the macroalkoxyamine is promoted, forming a 4-hydroxy-TEMPO stable free radical and a carbon-centered radical (in the isoprenic portion) in a way similar to the synthesis of IIR-*g*-MA. However, in this case, the carbon-centered radical can react with St and MA monomers to presumably produce first a block of alternating copolymer of St and MA [28]. Once the MA monomer is exhausted, excess styrene (which is present in a molar ratio of St/MA ≈ 19.3) can form a second block by the ensuing nitroxide mediated homopolymerization of styrene. Given the alternating nature of the MA–St copolymerization, the proposed structure of the resulting grafts (shown in Scheme 1) is thus poly[(styrene-*alt*-maleic anhydride)-*block*-styrene]. During the graft reaction, some increase of pressure in the reactor up to 103.4 kPa was observed; this was attributed to the vapor phase pressure from the reaction system (mainly toluene). At the end of the reaction the reactor was opened and two phases were observed: the soluble fraction was analyzed by ^1^H NMR; no evidence of IIR was found in this phase, but instead a “free polymer” of polystyrene homopolymer (due to the autothermal polymerization of St) and a copolymer of SMA, both chemically unlinked to the butyl rubber backbone, were identified. The insoluble fraction containing the graft polymer of IIR-*g*-SMA was isolated and recovered into methanol. The segregation was attributed to the poor solubility of the graft copolymer of IIR-*g*-SMA in the solvent. Finally, IIR-*g*-SMA was purified by re-dissolving as much of the material as possible in toluene and further precipitating it into methanol.

### 3.2. Spectroscopic Analysis

**Figure 1:**
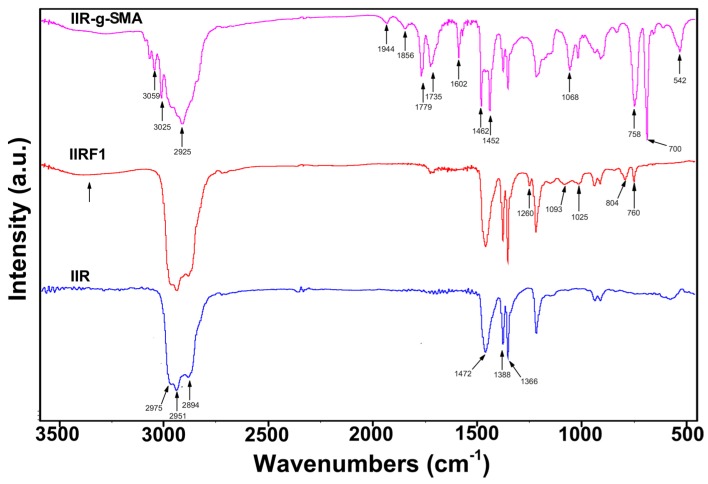
FTIR spectra of IIR (blue), IIRF1 (red), and LG IIR-g-SMA (pink).

Figure 1 shows the FTIR spectra of neat IIR, functionalized IIR with nitroxide moieties (IIRF1), and IIR-*g*-MA. Neat IIR showed characteristic symmetric and asymmetric stretching vibrations attributed to CH_2_ and CH_3_ (*ν_as_*_CH3_: 2951 cm^−1^, *ν_as_*_CH2_: 2875 cm^−1^, *ν_s_*_CH3_: 2894 cm^−1^). Also, bending vibrations of C–H were observed at: *δ_as_*_CH3_: 1472 cm^−1^, *δ_as_*_CH3_: 1388 cm^−1^, *δ_s_*_CH3_: 1366 cm^−1^. Functionalized IIR with OH-TEMPO moieties exhibited stretching vibrations *ν_s_* OH at 3398 cm^−1^ from OH-TEMPO. Furthermore, new vibrations attributed to piperidine ring of TEMPO were observed at 1260 and 1093 cm^−1^ [29], which were assigned to *ν_as_* C–N–C and C–N–(C)_2_, respectively. On the other hand, with respect to the grafted copolymer IIR-*g*-SMA (LG), its FTIR spectra show two bands in the carbonyl region corresponding to symmetric and asymmetric stretching of the conjugated anhydrides in the region of 1735 and 1779 cm^−1^, suggesting the presence of succinic anhydride in the modified IIR; the succinic groups also produce two bands due to stretching vibrations of the bond O=C–O–C=O, which can be identified with the bands at 1029 and 1068 cm^−1^. The presence of the aromatic ring from styrene can be confirmed with the overtones at 1859 and 1944 cm^−1^.

**Figure 2:**
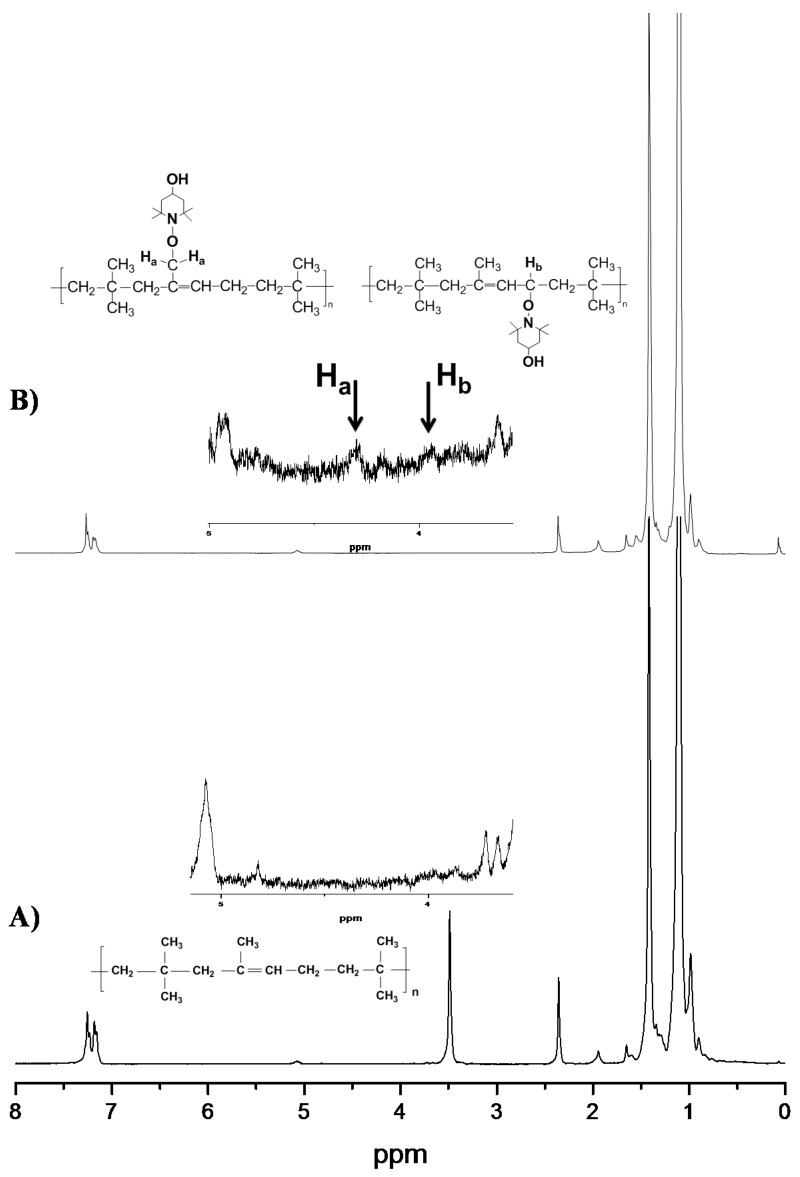
^1^H NMR spectra of (**A**) IIR and (**B**) IIRF1.

Unmodified polymer (IIR) and functionalized IIR with nitroxide/initiator (IIRF1 at lab scale and IIRF2 at a larger scale) were characterized and their structures investigated by ^1^H NMR (see Figure 2); however, since the abundance of the unsaturated moieties is much lower in the case of IIR than in the case of other polymers previously functionalized by us, the signals that can be ascribed to the attachment of the nitroxide moieties to the backbone are very weak and not conclusive.

Figure 2A shows the typical chemical shifts of IIR [30], which exhibits resonances centered at 5.06, 1.93 and 1.64 ppm attributable to the proton of methine –CH=, two protons of methylene –CH_2_– (alyllic to the double bond) and three protons of a methyl group –CH_3_, respectively, from the isoprenic portion. Resonances at 1.4 ppm and in the region of 1.1–0.97 ppm were also observed and attributed to two protons of methylene and 12 protons of four methyl groups respectively, attributed to the isobutylene fraction. Resonances centered at 3.5 ppm are attributed to methanol traces from the butyl rubber purification process (removal of antioxidants). It is also possible to observe a “clean zone” in the region of 4 ppm to 4.5 ppm in the spectrum. Figure 2B, corresponding to IIRF1, shows two new chemical shifts centered at 4.2 and 3.9 ppm, which could be attributed to the proton of the primary and secondary carbon attached to the nitroxide moiety, respectively, and are in good agreement with previous reports on the functionalization of several kinds of polymers with pendant nitroxide groups [18,20] and with simulations of the structures performed in the software ChemBioDraw Ultra (12.0), but these signals are rather weak and not conclusive, as noted above. Notice that compared with previous work in which, for example, polybutadiene was functionalized with TEMPO or its derivatives [18], in this case it was much more difficult to see the signals corresponding to the allylic protons of the rubber backbone in the 3.9–4.2 ppm region, since the abundance of these species is much lower in the case of IIR than in the case of polybutadiene; still, the signals were detected after several attempts of functionalization and NMR analysis under different conditions.

Finally, the spectra corresponding to IIR-*g*-MA produced at the lab scale show practically the same resonances observed for the functionalized IIR but, unfortunately, a peak that could be ascribed to a hydrogen in the succinyl moiety overlaps with hydrogen H_a_ in the IIRF1spectra, and hence the evidence is not conclusive (not shown here).

In spite of the difficulties found with the NMR analysis, in order to know the amount of MA present in the compatibilizer, an analysis by titration was performed. The MA ring was opened to obtain its corresponding acid, which was titrated using a 0.026 M solution of KOH/ethanol in the presence of phenolphthalein as indicator. The MA incorporation was found to be 1 wt %, which means that a 33% efficiency of functionalization was achieved.

### 3.3. GPC Analysis

**Figure 3:**
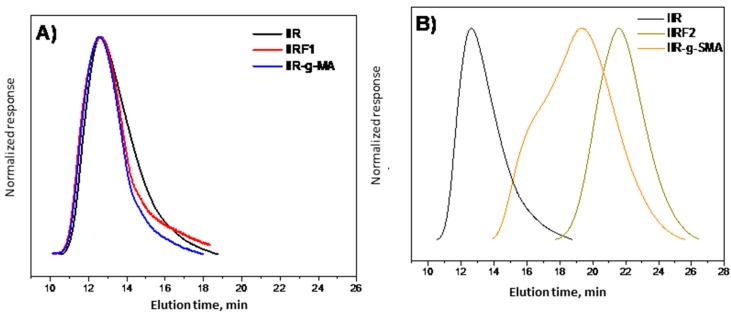
GPC of (**A**) IIR (*M*_n_ = 192,000 Da, PDI = 2.4), IIRF1 (*M*_n_ = 194,000 Da, PDI = 2.4), IIR-*g*-MA (*M*_n_ = 192,000 Da, PDI = 2.4); and (**B**) IIRF2 (*M*_n_ = 43,600 Da, PDI = 2.2), and IIR-*g*-SMA (*M*_n_ = 56,700, PDI = 2.75). The GPC of IIR is also included in Figure 3B.

In order to get more information on the functionalization process, analysis of the polymers via GPC was also performed. Figure 3 comparatively shows the gel permeation chromatograms of IIR, IIRF1, IIRF2, IIR-*g*-MA, and SG IIR-*g*-SMA. GPC analysis of the IIR-*g*-SMA resulted in *M*_n_ = 56,700, PDI = 2.8 for the SG type, and *M*_n_ = 64,300, PDI = 2.3 for the LG type.

Figure 3A, corresponding to GPC chromatograms of IIR, IIRF1, and IIR-*g*-MA, shows a slight shift of IIRF1 (194,000 Da, PDI = 2.4) towards higher molecular weights with respect to IIR, which is attributed to a change in the hydrodynamic volume of IIR (192,000 Da, PDI = 2.4) with nitroxide, providing further indication of the functionalization reaction. The average molecular weight of IIRF1 increased only slightly (~1%) with respect to that of IIR, and this smooth modification is attributable to the dropwise addition of the initiator during the functionalization process, which permits a certain control of the concentration of the *tert*-butoxi radical formed in the reaction. Further, IIR-*g*-MA exhibited practically the same distribution and elution time as IIRF1, which is a desirable feature in the synthesis of a compatibilizer, since it is well known that the maleation of olefin polymers by the extrusion process in the presence of a peroxide initiator, especially in the case of IIR, is detrimental to the molecular weight of the polymer [7,15]. By using the methodology proposed here, it is possible to preserve the molecular weight distribution (MWD) of the original IIR.

No broadening of the IIRF1 or the IIR-*g*-MA curves to low molecular weights is observed, indicating the absence of β-scission reactions; however, some broadening to higher molecular weight is detected. This can be due to limited branching reactions taking place between a radical formed along the backbone of one chain and an unsaturation in another chain [18,19,20].

Figure 3B, corresponding to GPC chromatograms from IIR, IIRF2, and IIR-*g*-SMA, shows the shift of IIRF2 (43,600 Da, PDI = 2.2) towards smaller molecular weights with respect to the virgin IIR. In this case, it is clear that chain degradation occurs during the functionalization process, opposite to the behavior observed in the functionalization of IIRF1. This is attributed to the different protocol of initiator addition used since in this case the total amount of initiator was dissolved together with the rubber, leading to the production of a large concentration of radicals during the heating period of the reaction. The radicals present in large concentration attack the unsaturations in the IIR chains, leading to β-scission and consequently to molecular weight reduction; as a result, the number-average molecular weight of IIRF2 decreases by 77% with respect to IIR. On the other hand, the grafted polymer IIR-*g*-SMA exhibits a shift towards high molecular weights with respect to IIRF2, together with an expected broad polydispersity, both attributed to the growth of grafts of SMA from the polymer backbone, which results in increased molecular weight. It is also possible to observe the overall reduction of the molecular weights during the functionalization process to form IIRF2, as well as the formation of a shoulder in the MWD after the grafting reaction.

### 3.4. Blend Compatibilization

#### 3.4.1. Izod Impact Strength

**Figure 4:**
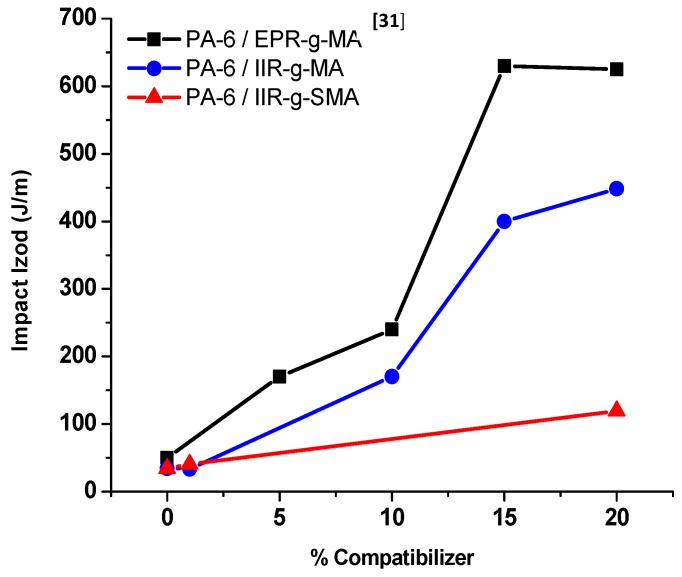
Comparison of the Izod impact strength of binary blends of IIR-*g*-MA and IIR-*g*-SMA with PA-6 as a function of total rubber content. A comparison is also made with data previously published for binary blends of PA-6 with ethylene-propylene rubber grafted with MA (EPR-*g*-MA) as modifier [31].

Model compatibilizers based on butyl rubber (IIR-*g*-MA and IIR-*g*-SMA), synthesized here, were tested in binary blends with Nylon-6 (PA-6), as described in the experimental section. The effect of the content of IIR-*g*-MA or IIR-*g*-SMA on room temperature Izod impact behavior is shown in Figure 4. In all cases, the points shown represent an average of 10 samples tested.

A noticeable toughening is achieved when a sufficient amount of IIR-*g*-MA is added (>10% of compatibilizer). The blends exhibited a toughness of 400 and 450 J/m using 15% and 20% of IIR-*g*-MA, respectively. For comparison, Figure 4 also shows the Izod impact strength of binary blends of PA-6/EPR-*g*-MA [31] at the same wt % content. The model compatibilizer IIR-*g*-MA synthesized here exhibits the same qualitative effect compared to a commercial compatibilizer based on EPR. On the other hand, the performance of a model compatibilizer of IIR-*g*-SMA in blends with PA-6 is poor, at least up to 20 wt % of the compatibilizer. This is mainly attributed to the poor miscibility of the polystyrene segments (presents in the SMA grafts) within the continuous phase of PA-6. Grafts of SMA on polypropylene have been proved effective in the compatibilization of poly(ethylene terephthalate)/(ethylene–propylene) (PET/EPR) blends due to the presence of the aromatic rings in PET [5].

**Figure 5:**
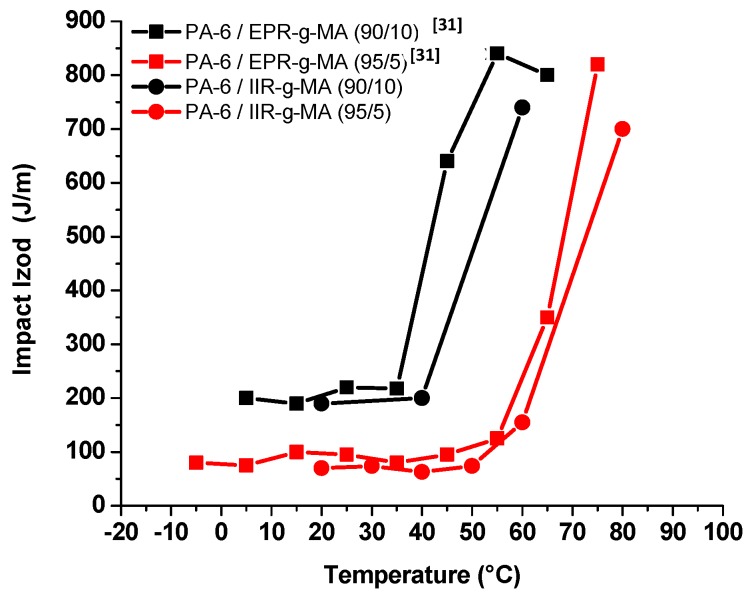
Izod impact strength as a function of temperature for binary blends at two IIR-*g*-MA contents (5% and 10%) where the continuous phase is PA-6. A comparison is also shown with data previously published for binary blends of PA-6 with EPR-*g*-MA as modifier [31].

Izod impact strength as a function of temperature for binary blends containing different wt % of IIR-*g*-MA prepared by twin screw extrusion is shown in Figure 5.

Blends with 5 wt % of IIR-*g*-MA show a ductile–brittle transition at a specific temperature of 50 °C. When increasing the amount of 10 wt % IIR-*g*-MA, it is possible to observe a shift of 10 °C in the ductile–brittle transition; in this case, the binary blends begin to show a ductile behavior at 40 °C. Thus, increasing the wt % of IIR-*g*-MA in the binary blend PA-6/IIR-*g*-MA produces a shift towards lower temperatures in the ductile–brittle transition. Also, in Figure 5, blends of PA-6/EPR-*g*-MA at 5 and 10 wt % of EPR-*g*-MA reported by Huang et al. [31] are presented in order to argue that the model compatibilizer IIR-*g*-MA synthesized here exhibits the same qualitative effect compared to a commercial compatibilizer based on EPR.

#### 3.4.2. Blend Morphology

TEM or STEM (SEM in transmission mode) can be used to characterize the morphology of polymer blends when one of the phases can be selectively stained in order to obtain good contrast [32]. However, these techniques generally only provide a 2D view of the blend morphology. The shape and size of rubber particles within polyamides has been extensively studied using software for processing images, assuming that the morphology is isotropic in three dimensions [32,33,34,35]. Software of this type usually identifies each individual particle forming the disperse phase and estimates its area, *A*, from which an apparent rubber particle size, *d*, can be calculated as follows:

(1) $$d={\left(4A/\pi \right)}^{1/2}.$$

**Figure 6:**
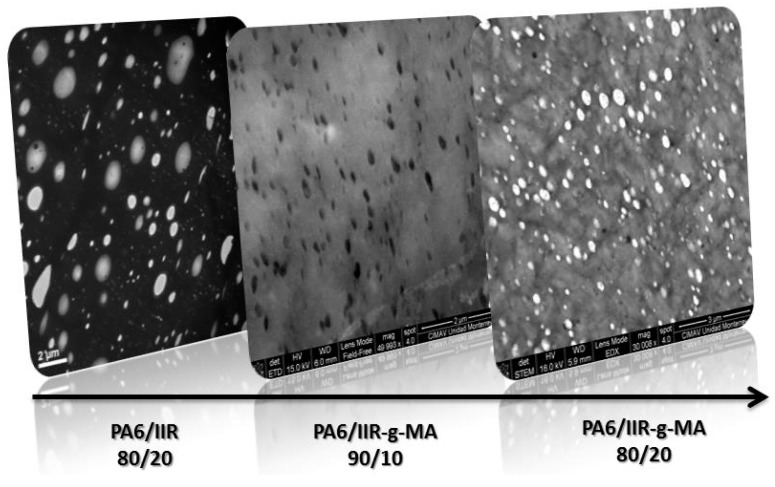
TEM photomicrographs of binary blends of PA-6/IIR (80/20 wt %), PA-6/IIR-*g*-MA (90/10 wt % and 80/20 wt %), prepared in a twin-screw extruder. Magnification used was 30,000×, 50,000×, and 30,000× respectively. The polyamide phase is stained dark with phosphotungstic acid. The central micrograph was taken in dark field since it was found that this modality resulted in better contrast.

Once the distribution of rubber particle sizes is obtained, the number, weight and volume average diameter values can be calculated as follows [31,36]:

(2) $${\bar{d}}_{n}=\frac{\sum n_{i}\times d_{i}}{\sum n_{i}}$$

(3) $${\bar{d}}_{w}=\frac{\sum n_{i}\times d_{i}^{2}}{\sum n_{i}\times d_{i}}$$

(4) $${\bar{d}}_{v}=\frac{\sum n_{i}\times d_{i}^{4}}{\sum n_{i}\times d_{i}^{3}}$$

where *n_i_* is the number of rubber particles with a specified diameter value *d_i_*. The weight average particle size, ${\bar{d}}_{w}$, has generally been used to relate toughness to particle size. Table 2 summarizes the rubber particle size analyses for binary blends (PA-6/IIR-*g*-MA) containing 10 and 20 wt % of IIR-*g*-MA, as well as for a blend PA-6/IIR (80/20, as control sample) and Figure 6 shows their corresponding micrographs by TEM or STEM.

Typically, the particle size increases with the rubber content (for blends prepared in a twin screw extruder) due to the fact that the probability of particle coalescence increases at higher rubber contents. Interestingly, binary blends using 10 or 20 wt % of maleated butyl rubber exhibited practically the same weight average particle size, ${\bar{d}}_{w}$. However, a more polydisperse system (polydispersity of the particle size given by ${\bar{d}}_{w}/{\bar{d}}_{n}$ or ${\bar{d}}_{v}/{\bar{d}}_{n})$ is obtained with an increasing wt % of compatibilizer. On the other hand, the “control sample” exhibited a rather large average particle size in comparison to the particle size obtained when a maleated rubber (at the same weight ratio) was used. This is due to the poor compatibility of the rubber with PA-6, as revealed by the TEM photomicrographs in Figure 6.

**Figure 7:**
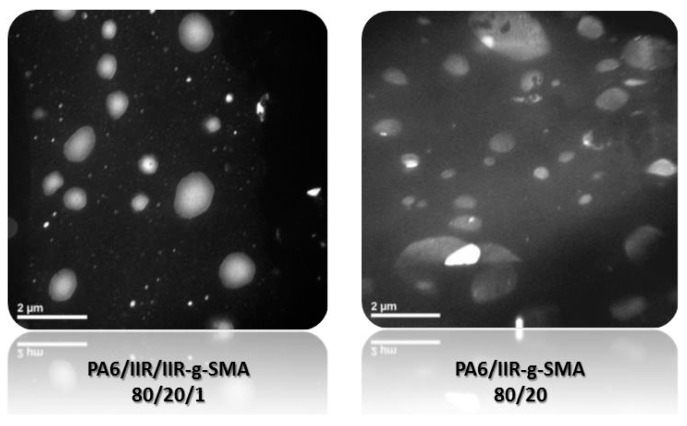
TEM photomicrographs of blends of PA-6/IIR/IIR-*g*-SMA (80/20/1 wt. ratio, **left**) and PA-6/IIR-*g*-SMA (80/20 wt %, **right**). Magnification used: 30,000×. The polyamide phase is stained dark with phosphotungstic acid.

Figure 6 shows morphological characteristics for binary blends of PA-6 with two IIR-*g*-MA contents (90/10 and 80/20 wt %) and the control sample PA-6/IIR (80/20 wt %). The rubber particles in this PA-6 matrix are quite spherical and regular in shape. Using this kind of compatibilizer based on butyl rubber, the rubber particle size does not seem to depend on rubber content (at least at 10 and 20 wt % contents) as shown in Table 2. Finally, Figure 7 shows TEM photomicrographs of blends of PA-6/IIR/IIR-*g*-SMA (80/20/1 wt. ratio) and PA-6/IIR-*g*-SMA (80/20 wt %) in order to test the performance of IIR-*g*-SMA as a compatibilizer in PA-6 blends.I

A binary blend of PA-6/IIR-*g*-SMA (80/20 wt %) shown in Figure 7 reveals rubber particles with high polydispersity, some of them exhibiting very large particle size (~2 μm), in comparison with blends having a similar wt % content of rubber but prepared using IIR-*g*-MA as compatibilizer (see Figure 6). In this case, an uneven particle size is obtained due to the low miscibility of the SMA grafts present along the backbone of the butyl rubber with PA-6; in particular, due to the polystyrene moieties present in the grafts of the IIR-*g*-SMA model compatibilizer.

A ternary blend of PA-6/IIR/IIR-*g*-SMA (80/20/1 wt. ratio) was also tested in order to diminish the amount of IIR-*g*-SMA and obtain in this way a smaller particle size. Based only on visual observation (not quantified), the particle size obtained was smaller with respect to that in blends PA-6/IIR-*g*-SMA (80/20 wt %), but slightly greater than that in blends PA-6/IIR-*g*-MA (80/20 wt %). The contribution of ~1 wt % of IIR-*g*-SMA in the blend PA-6/IIR was not significant in the compatibilization process. Furthermore, the presence of pure IIR in the ternary mixture (PA-6/IIR/IIR-*g*-SMA (ratio of 80/20/1 by weight)) leads to large particles of about 1 micron and tiny particles in the matrix (or continuous phase) of PA-6, similar to those observed in the mixture of PA-6/IIR-*g*-MA (80/20 wt %) shown in Figure 6, wherein the grafted material did not play the role of a compatibilizing agent. The presence of small amounts of IIR-*g*-SMA and the poor miscibility of the segments of SMA in the continuous phase of PA-6 can produce a segmentation in the particle size of IIR.

On the other hand, when a blend of PA-6/IIR-*g*-SMA (80/20 wt %) was performed, only large particles were observed due to the incompatibility or poor miscibility of SMA into the continuous phase.

### 3.5. Nanocomposite Preparation and Testing

Preliminary testing of IIR-*g*-SMA as a coupling agent in the preparation of IIR clay nanocomposites was done. Nanocomposite samples with 10 wt % of organo-clay were analyzed by STEM, revealing relatively good dispersion but incomplete exfoliation.

With respect to the mechanical properties, increasing the amount of nanoclay in the nanocomposite resulted in increased tensile strength with respect to the pure IIR. The samples in which SG IIR-*g*-SMA was used exhibited in general little effect of the nanoclay loading on the tensile resistance; the best results were obtained with an intermediate load. On the other hand, those samples in which LG IIR-*g*-SMA was used exhibited increased tensile resistance with an increasing nanoclay load. The samples in which some type of IIR-*g*-SMA was used showed lower tensile strength and higher elongation compared with those composites in which no coupling agent was used; however, all the nanocomposites exhibited increased tensile resistance compared to the pure IIR. This unsatisfactory performance of the IIR-*g*-SMA material as coupling agent is attributed to the deleterious presence of styrene in the grafts, which results in reduced compatibility with the matrix. The original idea of introducing styrene was to serve as an aid in the incorporation of additional MA in the grafts due to the practical impossibility of forming longer grafts of MA homopolymer; however, it seems that even small amounts of MA grafted as single units to the IIR backbone can be effective for nanoclay dispersion. This concept is being tested in our lab.

## 4. Conclusions

The procedure employed for nitroxide functionalization of butyl rubber using the unsaturations present in the isoprene units of IIR proved to be effective in spite of the relatively low content of isoprene in IIR. Once the nitroxide moieties were linked to the IIR backbone, further grafting from the functionalized rubber was possible by heating this material in the presence of maleic anhydride and (optional) styrene. The preferred procedure for nitroxide functionalization of IIR, if it is important to maintain the MWD of the rubber, involves the slow addition of a radical initiator in order to prevent the IIR from undergoing scission reactions, which would occur as a consequence of a high concentration of radicals from the initiator.

The modified IIRs were initially tested as impact modifiers/compatibilizers in blends with Nylon-6 and as coupling agents for the preparation of IIR-oganoclay nanocomposites.

Blends of PA-6/IIR-*g*-MA show a significant increase in impact resistance at increased loads of the modified rubber, as well as improved rubber particle dispersion in the nylon matrix.

On the other hand, when IIR-*g*-SMA is used as an impact modifier of PA-6, the improvement in impact resistance and rubber particle dispersion is marginal in spite of a larger amount of MA with respect to rubber used in the preparation of the modified IIR. Therefore, the use of styrene as an aid to increase the incorporation of MA in the grafts is not effective in the final application. Similarly unsatisfactory results occur when IIR-*g*-SMA is used as a coupling agent for the preparation of IIR/organoclay nanocomposites, in which incomplete nanoclay exfoliation is obtained. Despite an increase in tensile elongation observed for the nanocomposites that include IIR-*g*-SMA, the rest of the mechanical properties were not improved with respect to the samples without the modified rubber. Present work in our lab involves the preparation and testing of IIR-*g*-MA as a coupling agent for the exfoliation of nanoclays in an IIR matrix since, given the good results obtained with this material as a blend compatibilizer, it is expected that this grafted polymer shows good performance as a coupling agent.

## Figures and Tables

**Table 1:** PA-6/IIR, PA-6/IIR-*g*-MA, and PA-6/IIR-*g*-SMA (LG) blend conditions.

TAG	PA-6 (wt %)	IIR (wt %)	IIR-*g*-MA (wt %)	IIR-*g*-SMA (wt %)	Residence time, min	Temperature (°C)
B1	100	-	-	-	15	240
B2	80	20	-	-	15	240
B3	99	-	1	-	15	240
B4	90	-	10	-	15	240
B5	85	-	15	-	15	240
B6	80	-	20	-	15	240
B7	99	-		1	15	240
B8	80	-	-	20	15	240
B9	80 *	20 *	-	1 *	15	240

Note: * Only for B9 these quantities are not wt % but rather wt ratios.

**Table 2:** Rubber particle size comparison for binary PA-6/IIR-*g*-MA and PA-6/IIR blends.

IIR-*g*-MA (wt %)	IIR (wt %)	$\bar{d_{n}}$ (μm)	$\bar{d_{w}}$ (μm)	$\bar{d_{v}}$ (μm)	$\bar{d_{w}}/\bar{d_{n}}$	$\bar{d_{v}}/\bar{d_{n}}$
0	20	0.85	1.26	2.41	1.48	2.84
10	0	0.20	0.21	0.23	1.06	1.19
20	0	0.17	0.20	0.24	1.13	1.42
